# Daytime Changes in Tear Film Parameters and Visual Acuity with New-Generation Daily Disposable Silicone Hydrogel Contact Lenses—A Double-Masked Study in Symptomatic Subjects

**DOI:** 10.3390/vision8010011

**Published:** 2024-03-05

**Authors:** Rute J. Macedo-de-Araújo, Laura Rico-del-Viejo, Vicente Martin-Montañez, António Queirós, José M. González-Méijome

**Affiliations:** 1 Clinical & Experimental Optometry Research Lab (CEORLab), University of Minho, 4710-057 Braga, Portugal; aqp@fisica.uminho.pt (A.Q.); jgmeijome@fisica.uminho.pt (J.M.G.-M.); 2 Physics Center of Minho and Porto Universities (CF-UM-UP), 4710-057 Braga, Portugal; 3 Clinical and Experimental Eye Research Group, Faculty of Optics and Optometry, Universidad Complutense de Madrid, 28040 Madrid, Spain; lauraricodelviejo@gmail.com; 4 Department of Optometry and Vision, Faculty of Optics and Optometry, Universidad Complutense de Madrid, 28040 Madrid, Spain; 5 Optica Moncar, Lanzarote, Canarias, 35500 Arrecife, Spain; lz.vicen@gmail.com

**Keywords:** dynamic topography, daily disposable contact lenses, tear film stability

## Abstract

This prospective, double-masked, contralateral study aimed to analyze and compare daytime changes in pre-lens tear film (PLTF) stability and optical quality in symptomatic subjects wearing two contact lenses (CL). A secondary goal was to assess the performance of the PLTF by using dynamic topography techniques and analyzing surface asymmetry and irregularity indexes (SAI and SRI, respectively). Measurements were conducted on 20 symptomatic subjects (OSDI score > 13). Participants were fitted contralaterally and randomly with spherical Delefilcon A and Stenfilcon A CLs and underwent a series of measurements over 3 consecutive days: three in the morning (after 1–2 h of CL wear) and three in the afternoon (after 7–9 h of CL wear). High- and low-contrast visual acuity (HCVA and LCVA, respectively), pre-lens NIBUT, and dynamic topography were assessed. The contralateral fit of the two lenses allowed a direct and better comparison between them since they were exposed to the same conditions during the day. Consequently, both lenses demonstrated similar performance in HCVA, LCVA, and PLTF stability, with no statistically significant differences between them, although some fluctuations were observed throughout the day. Dynamic topography proved sensitive in evaluating temporal changes in the PLTF. The SRI index showed greater sensitivity to topographic changes due to lacrimal destabilization, making it potentially valuable for evaluating dry eye patients.

## 1. Introduction

The tear film plays a crucial role as an optical element and significantly contributes to proper visual function, serving as the eye’s primary refractive component. Fluctuations in the tear film’s dynamics can significantly impact the quality of vision and ocular surface-related symptoms [1,2]. Tear film dynamics after a blink are very variable and also depend on the interblink interval. It could remain stable, present local reductions in thickness, or present complete breaks (tear break-up). The local variations that can occur in tear film thickness will induce aberrations in the optical system. If variations in tear film thickness occur over the pupil area, it will lead to a reduction in retinal image quality and an overall degradation of visual quality [3,4,5]. Therefore, a smooth and regular tear film over the pupil area is important to obtain high-quality retinal images [3]. The optical quality after a complete blink changes a lot over time. In normal eyes, there is a trend toward a reduction in corneal aberrations right after a blink, which corresponds to a phase of lacrimal reorganization known as tear build-up time. There is a gradual increase in optical aberrations a few seconds after the tear break-up, which will lead to a progressive deterioration of the optical quality of the eye (mean decrease of 21 ± 8%) [2]. The aberrations seem to be lowest 6 s after a complete blink, so it is difficult to detect the effects on visual quality when the interblink interval is about 4 s [6]. 

Contact lenses (CLs) perturb the stability of the tear film. Inserting a CL divides the tear film into two layers: the pre-lens tear film (PLTF) and the post-lens tear film (PoLTF). Changes include decreased tear meniscus height with CL wear [7] and attenuation of the tear lipid layer spread, limited wettability of the CL surface, and increased friction with the eyelid wiper [8]. These are also accompanied by other alterations that will influence the overall optical quality and comfort and may result in symptoms of CL discomfort (CLD) [9,10,11]. CLD is multifactorial, either related to the CL (material, design, and care) and/or to the environment (compliance, ocular surface conditions, and patient-specific factors) [11], with dryness symptoms being the most common complaints of CL wearers. All these factors contribute to the increased discontinuation of CL wear [12,13]. Therefore, it is of paramount importance to evaluate the tear film before CL fitting and at subsequent follow-up evaluations [14]. Various studies confirmed the deterioration of PLTF stability with different types of CL in comparison to pre-corneal tear film stability [15,16,17,18,19,20]. Apart from CL wear, some studies state that tear film dynamics will differ throughout the day, with decreased values of tear break-up time (TBUT) being found after awakening [21] and at the end of the day [22], although other authors did not find any difference throughout the day [23,24]. Studies have shown reduced TBUT values in females and the elderly [25,26,27], with greater temporal changes in females [28]. Irregularities resulting from refractive surgery and ocular/palpebral surgery will also impact tear film dynamics [29]. It is also important to consider the environmental conditions that will have an impact on tear film stability (temperature, humidity, air conditioning, and pollution) [29]. 

The main goal of the present study was to analyze and compare the daytime changes in PLTF stability and optical quality over 3 consecutive days with two daily disposable CLs fitted contralaterally (exposed to the same environmental conditions). A secondary goal was to study the behavior of the PLTF during a short period of time without blinking using dynamic topography techniques by analyzing the surface asymmetry and irregularity indexes (SAI and SRI).

## 2. Material and Methods

### 2.1. Study Design and Subjects

This was a prospective, double-masked, contralateral study that intended to compare two different daily disposable CLs with respect to their PLTF stability and optical quality during the day and over different days. Both wearers and examiners were blinded to the lens material and brand. The study employed a contralateral fitting approach, assessing two different lenses worn simultaneously by the same subject, with random assignment of the lens material for each eye. Sample size calculations (https://hedwig.mgh.harvard.edu/sample_size/size.html, accessed on 1 January 2013) revealed the need to include 18 patients, considering a power of 0.8 and a 0.05 significance level to detect a 1.5 unit difference in tear break-up time. Twenty symptomatic subjects were recruited (13 female, 7 male) with a mean age of 26.75 ± 6.28 years. Inclusion criteria comprised participants aged between 18 and 40 years, who must be CL wearers, even occasionally (more than 3 days per week), and must have symptoms of discomfort either with and/or without CL wear (Ocular Surface Disease Index questionnaire (OSDI) > 13) that is equal in both eyes (i.e., subjects with symptoms in one eye only were excluded). Subjects with ocular opacities, history of ocular surgery or disease, or subjects taking any medications with ocular affection were excluded. Inclusion criteria comprised subjects with a best corrected VA of 0.00 logMAR units or better and a refractive cylinder below 1.00 D. The difference of VA between both eyes must be of less than 0.1 logMAR units and have less than 1.00 D of anisometropia. 

### 2.2. Contact Lenses

The daily disposable CLs used were Delefilcon A (Dailies Total1—Alcon, Geneva, Switzerland), with a water gradient that transitions from 33% water content at the lens core to more than 80% at the lens surface, and Stenfilcon A (Myday—CooperVision, San Ramon, CA, USA), which incorporates a new chemical structure (SmartSilicone^TM^) that enables efficient channels for oxygen delivery to the cornea. Stenfilcon A lenses require less silicone in their material to achieve the desired oxygen permeability, which has a direct impact on the lens wettability and modulus of elasticity (Smart Silicone technology). More specifications of the lenses are shown in Table 1. The lenses were fitted contralaterally, that is, one on the right eye and one on the left eye, randomly selected. Subjects were randomly fitted with Delefilcon A or Stenfilcon A on the right or left eye. Randomization was applied for all three days of measurements, so when the subjects were wearing different lenses throughout the days in each eye, neither the subjects nor the clinician was aware of the lens that was fitted on each day (double-masked). 

### 2.3. Clinical Examination Routine

In the baseline visit (V0), the subjects underwent a full optometric examination, which included anamnesis, an Ocular Surface Disease Index (OSDI) questionnaire, refraction, logMAR high-contrast distance visual acuity (HCVA), topography, noninvasive tear break-up time (NIBUT) with Tearscope (Keeler, Windsor, UK), and slit-lamp examination. V0 was performed in the morning (between 9 a.m. and 12 p.m.), and the subjects were instructed not to wear their habitual lenses the 2 days before. An informed consent form was signed before enrollment. 

The lens blisters were masked and the randomization process (https://www.randomizer.org/#randomize, accessed on 1 January 2013) was carried out by a third person so that neither the subject nor the examiner knew which lens the subject would be wearing in each eye. The subjects were instructed to use the lens assigned with *“OD”* in the right eye and *“OE”* (OS in Portuguese) in the left eye. All subjects agreed to attend the 6 visits on 3 consecutive days according to the protocol, during a study period of 6 days. Day 1—24 h of *“wash-out”* period (no CL wear); Day 2 and Day 3—wear of the first pairs of CLs assigned for each eye provided by the examiner for more than 8 h, without attending any examination; Day 4, Day 5, and Day 6—subjects should wear the CLs and attend the scheduled visits: one in the morning (1 to 2 h after lens insertion) and the other in the afternoon (7 to 9 h of CL wear). Measurements of the study were completed on Day 4 (V1 and V2), Day 5 (V3 and V4), and Day 6 (V5 and V6). The same parameters were evaluated in all visits.

### 2.4. Comfort Assessment

The Ocular Surface Disease Index (OSDI) questionnaire was employed for the subjective assessment of symptoms, as well as for the characterization and selection of the sample [30,31]. The OSDI questionnaire was selected over other CL-related questionnaires (like CLDEQ-8) because some participants were sporadic CL wearers because of their discomfort. As the OSDI questionnaire did not directly relate to CL wear but to the overall sensations of the subjects, the discomfort score during a “normal” week for all subjects could be assessed. This is a 12-item questionnaire designed for the assessment of dry eye symptoms and visual-related functioning. The OSDI has good to excellent reliability, validity, sensitivity, and specificity [32]. The total OSDI score was calculated on the basis of the following formula: OSDI = [(sum of scores for all questions answered) × 100]/[(total number of questions answered) × 4]. This was built on a 0 to 100 scale, with higher scores indicating greater disability. A cutoff value of 13 of the OSDI score was used to group patients into asymptomatic and symptomatic, as suggested by previous studies [10,31,32]. 

### 2.5. Visual Acuity

High-contrast (100%) visual acuity (HCVA) and low-contrast (10%) visual acuity (LCVA) were measured with the Logarithmic Visual Acuity Chart ETDRS (Precision Vision, Woodstock, IL, USA) at 4 m. This was assessed in all the visits (from V0 to V6) monocularly and binocularly, with best spectacle correction (V0) and with the assigned lenses (V1 to V6). 

### 2.6. Noninvasive Tear Break-Up Time (NIBUT)

Noninvasive tear break-up time (NIBUT) was assessed with Tearscope (Keeler, Windsor, UK) with the NIBUT grid, which allows the evaluation of the tear layer with minimal impact on tear dryness because of its cold cathode light. The patient was asked to open the eye for the maximum time possible until the observer saw the rupture. The time between the last blink and the appearance of the first distortion in the lines was registered (break-up time) using the instrument’s stopwatch. Three measures were performed in each eye in each one of the visits. The location of the first rupture was also noted considering the 5 principal corneal zones: 1—central; 2—nasal; 3—temporal; 4—superior; and 5—inferior. It is important to emphasize that the measurements were always performed by the same observer.

### 2.7. Dynamic Topography

Dynamic topography with the lens in situ was obtained from a corneal topographer (Medmont E300, Medmont, Nunawading, Australia). A total of 11 images over 10 s—from 0 (right after the last complete blink) to 10 s—were selected. The eye should remain open throughout the 10 s of measurements, and it was repeated if the patient blinked. The irregularities caused by tear film disruption can distort the topographic images and can be quantified by the indexes SAI (surface asymmetry index) and SRI (surface regularity index) [33]. The SAI is the global measure of corneal asymmetry, with sensitivity to detect off-center corneal ectasias by comparing areas of the cornea that are 180 degrees apart on the same chord. As such, centrally located cones (central keratoconus) and regular astigmatisms will not impact the SAI, contrary to decentered cones and irregular astigmatism [34,35]. SAI values between 0.10 and 0.42 are considered normal [35]. The SRI analyzes and quantifies dioptric powers of adjacent points in 256 hemi-meridians in the 10 central rings, [34] characterizing local corneal power fluctuations over the central 4.5 mm chord area. The SRI can predict the optical outcome that might be expected based on corneal topography (central corneal optical quality), with normal values ranging from 0.0 to 0.56 [35,36]. In summary, the SAI and SRI represent variations in corneal contours and can provide information about the relation between the corneal and tear film status [37]. It was hypothesized that in the present study, the SRI could be more sensitive to detect tear film break-up points due to its local specificity. For a better understanding of the assessments, the TSRI (difference between the maximum and minimum SRI values at each measure) and TSAI (difference between the maximum and minimum SAI values at each measure) were also assessed and discussed.

### 2.8. Statistical Analysis

Statistical analysis has been conducted using SPSS v22.0 (IBM Inc., Chicago, IL, USA). The descriptive data are presented in terms of mean ± standard deviation (SD). The normality of all variables was evaluated using the Shapiro–Wilk test. For comparisons between the visits, the comparison of means was analyzed using ANOVA and Friedman’s test if the variable presented a normal or non-normal distribution, respectively. Pairwise comparisons were conducted with a paired samples *t*-test or a Wilcoxon test, considering the normality distribution of the variables in the analysis. The level of statistical significance has been set at *p* < 0.05.

## 3. Results

### 3.1. Sample Characteristics—Baseline Results

Table 2 shows the demographic characteristics of the sample, as measured during the baseline visit (V0). The mean OSDI score was 32.95 ± 9.82 (range: 16.67 to 56.25), evidencing the degree of symptomatology of the participants. 

### 3.2. Visual Acuity

Table 3 shows the results for monocular HCVA and LCVA. For Delefilcon A lenses, there were no statistically significant differences between the different morning visits for HCVA (V1, V3, and V5), contrary to afternoon visits (*p* = 0.021, Friedman), with a mean difference between Day 1 and Day 3 (*p* = 0.015, Wilcoxon) of 2.5 letters. For LCVA, there were statistically significant differences only for afternoon visits (*p* = 0.043, Friedman), with differences lower than 2 letters between visits. As shown, there is a slight improvement from Day 1 to Day 3 in both HC- and LCVA for both morning and afternoon visits. For Stenfilcon A lenses, there was only one statistically significant difference between the morning and afternoon visit on Day 1, with a better LCVA at the end of the day (*p* = 0.024, paired sample *t*-test). For binocular vision, there was also a statistically significant difference in Day 1 for LCVA. No statistically significant differences between the two lenses were found. 

### 3.3. Pre-Lens NIBUT

The pre-lens NIBUT values measured with Tearscope are shown in Table 4. No statistically significant differences were found between the morning visits and between afternoon visits for any of the lenses studied (*p* > 0.05). Time comparisons (morning vs. afternoon) revealed a decrease in NIBUT in the afternoon for Stenfilcon A (*p* > 0.05), but this difference was statistically significant only on Day 3 for Delefilcon A (*p* < 0.05). It is important to highlight that the decrease in NIBUT from the morning to the afternoon was less than 1 s in both lenses.

**Figure 1:**
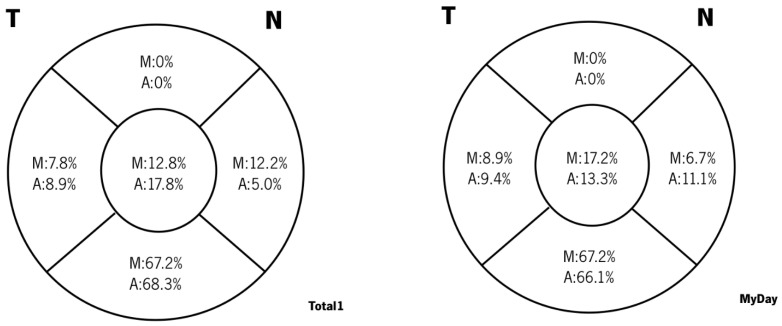
Location of the first tear film disruption in Delefilcon A lens (**left**) and Stenfilcon A lens (**right**). Results are expressed in percentage. M: morning visits; A: afternoon visits.

The localization of the initial tear film disruption in the morning and afternoon visits is illustrated in Figure 1. Tear film breakage predominantly occurred in the inferior zone the majority of the time. This observation is true for both lenses and for both morning and afternoon visits. Notably, tear disruption never occurred in the superior zone. 

### 3.4. Dynamic Topography

**Figure 2:**
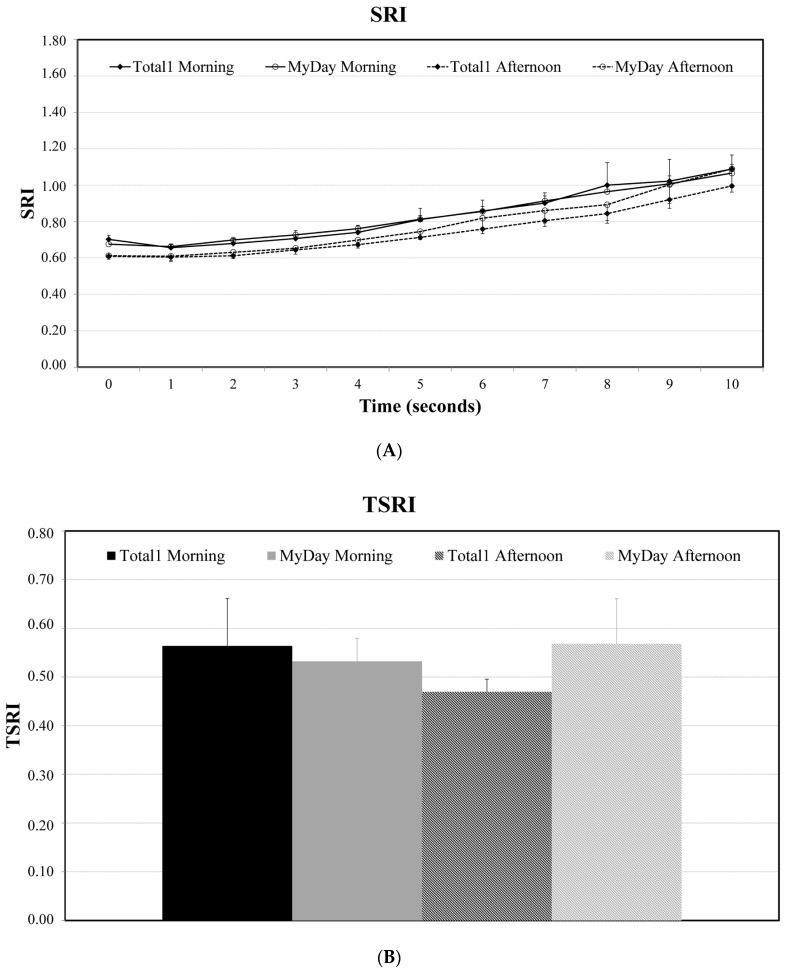
Differences in SRI (**A**) and TSRI (**B**) in morning and afternoon visits between the two lenses.

As there were no statistically significant differences between the three morning visits and the three afternoon visits for any of the lenses, the mean of the three morning visits and the three afternoon visits will be presented in order to compare the two lenses. Figure 2A presents the mean of the three morning visits (line) and the three afternoon visits (dashed line) for both lenses. The two lenses promoted a similar behavior in the morning visits. By the afternoon, Stenfilcon A showed higher values on the SRI, although there were no statistically significant differences between the two lenses, either in afternoon or morning visits. The SRI values became higher the longer the eye was open, suggesting a more irregular surface, with the increases being after 2 s of open eye condition. Analyzing TSRI values, Stenfilcon A lenses promoted a better performance in the morning, but it was much better for Delefilcon A in the afternoon. Delefilcon A seems to improve its surface stability during the day when compared to Stenfilcon A, although there were no statistically significant differences between them. 

**Figure 3:**
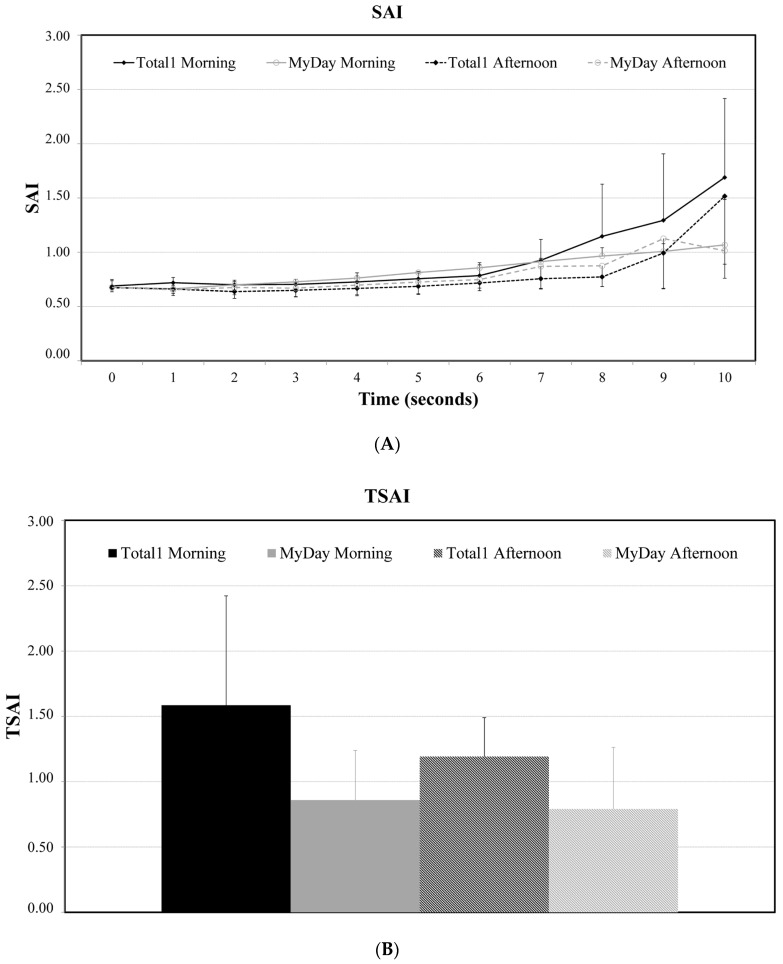
Differences in SAI (**A**) and TSAI (**B**) in morning and afternoon visits between the two lenses.

The SAI values are presented in Figure 3A. The SAI values were higher for Delefilcon A lenses by the morning when compared to the mean of the same visits for Stenfilcon A lenses. By the afternoon, the results were the opposite, with Delefilcon A showing lower values until 9 s. These results suggest that Delefilcon A has a worse performance in the morning and is better in the afternoon when compared to the Stenfilcon A lens. Despite this, there were no statistically significant differences between the two lenses. TSAI values revealed better performance for the Stenfilcon A lens, but there were no statistically significant differences between the two lenses. 

## 4. Discussion

The performance of two daily disposable CLs fitted contralaterally was compared in the present study. Regarding visual performance, no differences were observed between the two lenses in both HCVA and LCVA, although some fluctuations were noted throughout the day. A study from Belda-Salmerón et al. [38], which also intended to evaluate both HCVA and LCVA in intervals of 2 h during 1 day of lens wear, showed that the greatest differences between lenses were obtained in LCVA. Similarly to the findings of the present study, Delefilcon A CLs achieved a better performance. Another study that intended to compare three daily disposable CLs did not find statistically significant differences between them, with values ranging from −0.12 to −0.14 for HCVA and 0.13 to 0.19 for LCVA—differences comparable to those found in the present study [39]. Considering that the participants of the study were all successfully fitted with their CLs, the minimal VA fluctuations and minimal differences between both lenses could be attributed to the differences in the inherent properties of the two lenses, such as the material and water content [38]. HCVA may not be a metric sensitive enough to reveal the subtle changes in visual performance. Although no statistically significant correlations were found, VA was very stable from morning to afternoon, with minimal clinically insignificant differences of 1–2 letters of VA. The same trend happened with NIBUT, with the morning–afternoon differences being no higher than 0.8 s. However, greater changes/decreases in HCVA and NIBUT were found in the Stenfilcon A lens. Although the clinical significance can be neglected, there seems to be a cause–effect here in which the greater tear destabilization caused by this lens influences the LCVA. For example, Day 1 is the day on which the NIBUT decreases the most and also coincides with the day on which the LCVA decreased by 2 letters.

The presence of a CL destabilizes the tear film. Some studies have established a cutoff value of TBUT < 10 s and NIBUT < 10 to indicate abnormal tear film stability (pre-corneal tear film) [40]. In the present study, the mean baseline NIBUT was below 10 s in both eyes, indicating tear film destabilization in the recruited subjects. Analyzing tear film stability over time with CL wear revealed a reduction in pre-lens NIBUT from morning to afternoon across all three days. These changes were more pronounced with the Stenfilcon A lens, showing statistically significant differences; however, the mean decrease was less than 1 s on all three days. With the Delefilcon A lens, a statistically significant difference between morning and afternoon pre-lens NIBUT was only observed on Day 3 (mean difference of 0.74 ± 1.58 s). No statistically significant differences were found between the two lenses analyzed in the present study. Despite this, a previous study found that Delefilcon A had a higher NIBUT over the lens than the other two lenses tested (Filcon-II and Narafilcon A) [41]. The reduction in NIBUT at the end of the day was previously mentioned in the literature. Lira et al. [22] found a statistically significant reduction in NIBUT during the day of about 1.2 s in non-CL wearers. In another study [42], a reduction of 0.1 s was observed in asymptomatic CL wearers and 2.55 s in the symptomatic ones after 5 h of CL wear. Although statistically nonsignificant, the present findings suggest that Delefilcon A lenses may have a lesser impact on the stability of PLTF throughout the day compared to Stenfilcon A lenses. Wolffsohn and colleagues [43] have examined the clinical performance of daily disposable CLs over 16 h and found that the pre-lens NIBUT decreased namely after 8 h of lens wear. Others concluded that the tear film of intolerant CL wearers changed less than asymptomatic patients, potentially because the first ones have pre-existing tear film defects, such as lower tear volume and a more destabilized tear film [44]. This may justify the small differences observed during 1 day of lens wear (<1 s), as all the patients were symptomatic and may have had pre-existing tear defects. Although it was not possible to categorize them as having low tear volume, they certainly experienced issues related to the tear dynamics at the front ocular surface, as evidenced by our tear stability results.

The dynamic topography results are expressed with the SRI and SAI values like in previous studies [33,45], and with the respective calculation of TSRI and TSAI values. Analyzing SRI, no statistically significant differences between lenses were observed, with both showing very similar behavior. Stenfilcon A lenses showed slightly higher values on the SRI in the afternoon, possibly depicting a more destabilized tear film. Aligned with that, the analysis of TSRI revealed a more unstable tear film for Stenfilcon A in the afternoon, but Delefilcon A was more unstable in the morning visits. The analysis of SAI values also revealed no statistically significant differences between lenses. Analyzing the behavior of both CLs, Delefilcon A revealed the worst performance in the morning and better performance at the end of the day up to 8 s but then worsened significantly up to 10 s. Aligned with this, the TSAI values also confirmed the worst performance for Delefilcon A in the afternoon visits and surprisingly in the morning as well, as TSAI is the mean difference between the maximum and minimum SAI value at each measure. Through direct observation of the figures, one can conclude that the SRI seems to be a more sensitive metric to tear film dynamics than the SAI, evidencing more oscillations that could be related to tear film disruption. SAI values only begin to increase 5–6 s after a blink, while SRI values start worsening in the first seconds after a complete blink. Observing Table 4, it is noticeable that pre-lens NIBUT values range from 4.8 to 6 s (approximately). In this sense, the SRI was more sensitive to detecting the local variations (changes in tear film thickness) that occur moments after a complete blink, even before a complete break-up of the tear film. On the other hand, SAI values were only majorly affected after the tear break-up. Previous works already reported a more stable tear film at the end of the day when compared to the morning, with systematic improvements during the day [46]. The results of the present work revealed a more stable performance in SAI values (stable up to 7 s after a complete blink), contrary to the destabilization found in SRI values right after the blink. Iskander et al. [47] also found that SAI values could be very stable up to 12 s after a blink. It is known that dry eye patients have increased SAI, TSAI, SRI, and TSRI values compared to healthy subjects [37]. In the present study, with symptomatic subjects, the SRI ranged from 0.60 to 0.70 right after a complete blink and reached values between 1.00 and 1.09 after 10 s—values significantly higher than those reported in healthy eyes by other studies [37,48]. However, one of those studies also evaluated a group of dry eye patients and found values ranging from 1.5 to almost 2, which are higher than the ones found in the present study [37]. Also, in the present study, the minimum values were found within the first 2 s after a blink; however, others found minimum values of the SRI at 7 s, which was attributed to the rearrangement of the tear film known as tear build-up [33]. However, it is important to highlight that those measurements were carried out without CLs, reflecting the different dynamic behavior of the tear film when a CL is placed on an eye where there is not a marked tear build-up, as the minimum SRI value was found after 1 s of open eye conditions. This is also true for SAI values, with the minimum value being found at 0 s in the present study and at 5.43 ± 2.72 s in previous reports [33]. In the study conducted by Kojima et al. [37] where subjects with healthy eyes and subjects with dry eyes were evaluated, different results were also found. In the healthy eye group, the values of SAI were very consistent through the 10 s of measurements, contrary to the behavior in the dry eye group, with values ranging from 1.5 to 2.6. In the present study, SAI values revealed a stable behavior up to 8 s of open eye, followed by a significant increase. It is difficult to directly compare these results with Németh et al. [48] as the authors evaluated non-CL wearers. Taking this into account, their outcomes revealed more stable values when compared to the present results. The mean TSRI and TSAI values were higher in the present work, supporting that CL wear can have some role in tear film destabilization. 

In general, the SAI and SRI values were higher than normal cornea values, potentially due to tear film destabilization caused by CL presence and irregularities of the anterior surface of the CL. Another noteworthy observation is that SRI values increased shortly after 2 s following a complete blink, while SAI values remained stable until around 7 s before beginning to increase. Given that SAI values are more indicative of peripheral changes and SRI values of central changes, we can infer that PLTF degradation begins in the central area approximately 2 or 3 s after a complete blink, with peripheral effects occurring later. However, it is crucial to note that reported tear break-up positions were predominantly in the inferior zone (>60% of the time in both lenses). Nevertheless, it is important to consider that these observations are based on visible rupture points observed by the examiner using Tearscope. SRI values may reveal tear film destabilization before it becomes detectable through direct observation. 

## 5. Conclusions

The contralateral fit of the lenses allowed a direct and better comparison between them, as they were exposed to the same conditions throughout the day. As a result, both lenses exhibited similar performance in HCVA, LCVA, pre-lens NIBUT, and dynamic topography (SAI and SRI values). Dynamic topography, particularly SRI values, proved to be sensitive in the assessment of the temporal changes in the PLTF. SRI values were found to be more sensitive to changes in the tear film dynamics over time, indicating variations possibly related to local thinning of the tear film. Thus, SRI could serve as a better index for objectively assessing tear film dynamics, with numerous applications in evaluating dry eye disease and categorizing its severity.

## Figures and Tables

**Table 1:** Parameters of the contact lenses used in the study.

Lens	Material	Diameter (mm)	Base Curve (mm)	Centre Thickness (mm)	Water Content (%)	DK/t	Modulus (MPa)
**Total1**	Delefilcon A	14.1	8.5	0.09	>80% at surface 33% at core	156	0.7
**MyDay**	Stenfilcon A	14.2	8.4	0.08	54%	100	0.4

**Table 2:** Demographic characteristics of the participants recruited.

Parameter	Description
N	20
Gender	13 F (65%)
	7 M (35%)
Age (years)	26.75 ± 6.28
	26.92 ± 6.4 F
	26.42 ± 6.6 Ma
Habitual refraction (D) Mean ± SD	Pre-Delefilcon A Eye
	−1.97 ± 1.14 DS
	−0.11 ± 0.27 DC
	Pre-Stenfilcon A Eye
	−1.88 ± 1.15 DS
	−0.16 ± 0.33 DC
Keratometry Mean ± SD	Pre-Delefilcon A Eye: 7.71 ± 0.26 (D)
	Pre-Stenfilcon A Eye: 7.79 ± 0.26 (D)
Habitual correction	Glasses and sporadic CL wear: 5
	Sporadic CL wear only: 2
	Daily disposable CL wear: 5
	Monthly CL (daily wear): 8
NIBUT (seconds) Mean ± SD	Pre-Delefilcon A Eye: 7.73 ± 2.2 s
	Pre-Stenfilcon A Eye: 8.3 ± 2.9 s
OSDI Mean ± SD	32.95 ± 9.82 (range: 16.67 to 56.25)
	33.36 ± 9.7 F
	32.70 ± 10.9 Ma

F, female; M, male; M, equivalent sphere; Pre-Delefilcon A Eye, eye that wore Delefilcon A lens by randomization; Pre-Stenfilcon A Eye, eye that wore Stenfilcon A lens by randomization; DS, diopters of sphere; DC, diopters of cylinder. Age, refraction, NIBUT, and OSDI are expressed as means ± SD.

**Table 3:** Monocular and binocular high-contrast visual acuity and low-contrast visual acuity (HCVA and LCVA, respectively) in LogMAR scale for the Delefilcon A and Stenfilcon A lenses. Results are expressed as mean ± SD.

		HCVA					LCVA		
		Day 1	Day 2	Day 3	Mean p (a)	Day 1	Day 2	Day 3	Mean p (a)
Delefilcon A	Morning	−0.05 ± 0.07	−0.05 ± 0.10	−0.07 ± 0.11	−0.06 ± 0.08 0.821 *	0.17 ± 0.10	0.16 ± 0.11	0.13 ± 0.12	0.16 ± 0.10 0.081 +
	Afternoon	−0.03 ± 0.10	−0.05 ± 0.10	−0.08 ± 0.10	−0.05 ± 0.09 **0.021** +	0.17 ± 0.12	0.14 ± 0.10	0.13 ± 0.12	0.15 ± 0.10 **0.043** +
	Difference p (b)	−0.02 ± 0.06, 0.144 º	0.00 ± 0.07, 0.984 ×	0.01 ± 0.06, 0.425 º		0.00 ± 0.07 0.950 º	0.02 ± 0.06, 0.079 º	0.00 ± 0.06, 0.801 º	
Stenfilcon A	Morning	−0.04 ± 0.09	−0.04 ± 0.11	−0.06 ± 0.11	−0.04 ± 0.09 0.771 *	0.17 ± 0.09	0.15 ± 0.12	0.12 ± 0.09	0.15 ± 0.08 0.196 *
	Afternoon	−0.05 ± 0.10	−0.05 ± 0.10	−0.06 ± 0.09	−0.05 ± 0.09 0.877 *	0.14 ± 0.11	0.12 ± 0.08	0.12 ± 0.11	0.13 ± 0.09 0.766 *
	Difference p (b)	0.01 ± 0.06 0.468 º	0.01 ± 0.08 0.484 º	0.00 ± 0.08 0.769 º		0.04 ± 0.06 **0.024** º	0.04 ± 0.12 0.304 ×	0.00 ± 0.05 0.729 º	
BINOCULAR	Morning	−0.15 ± 0.17	−0.12 ± 0.07	−0.14 ± 0.08	−0.14 ± 0.09 0.513 +	0.09 ± 0.06	0.06 ± 0.07	0.04 ± 0.05	0.06 ± 0.05 0.068 *
	Afternoon	−0.12 ± 0.08	−0.13 ± 0.06	−0.12 ± 0.08	−0.13 ± 0.07 0.921 *	0.06 ± 0.06	0.05 ± 0.05	0.05 ± 0.06	0.05 ± 0.05 0.461 +
	Difference p (b)	−0.02 ± 0.16, 0.636 ×	0.01 ± 0.04, 0.280 º	−0.01 ± 0.07, 0.452 ×		0.03 ± 0.05, **0.029** º	0.01 ± 0.05, 0.267 º	−0.01 ± 0.04, 0.391 º	

Statistically significant differences between the groups are presented in bold; p (a), difference between the three visits: (*) ANOVA; (+) Friedman. p (b): difference between morning and afternoon visits: (º) paired sample *t*-test; (×) Wilcoxon.

**Table 4:** Monocular pre-lens NIBUT for Delefilcon A and Stenfilcon A lenses, measured over 3 days in the morning and afternoon. Results are shown as mean ± SD.

		Day 1	Day 2	Day 3	Mean (s) p (a)
Delefilcon A	Morning	5.94 ± 1.58	5.27 ± 1.33	5.57 ± 1.31	5.59 ± 1.05 0.387 +
	Afternoon	5.21 ± 0.82	4.80 ± 0.86	4.83 ± 1.17	4.95 ± 0.63 0.326 *
	Difference p (b)	0.73 ± 1.63 0.102 ×	0.47 ± 1.22 0.101 º	0.74 ± 1.58 **0.048** ×	
Stenfilcon A	Morning	5.97 ± 1.58	5.45 ± 1.15	5.66 ± 1.43	5.69 ± 1.21 0.165 +
	Afternoon	5.07 ± 0.79	4.87 ± 1.32	4.93 ± 1.95	4.96 ± 0.93 0.200 +
	Difference p (b)	0.89 ± 1.29 **0.006** º	0.58 ± 0.99 **0.017** º	0.74 ± 1.90 **0.007** ×	

Statistically significant differences between the groups are presented in bold; p (a), difference between the three visits: (*) ANOVA; (+) Friedman. p (b): difference between morning and afternoon visits: (º) paired sample *t*-test; (×) Wilcoxon.

## Data Availability

The raw data supporting the conclusions of this article will be made available by the authors on request.
